# Sample content of kinesthetic educational training: Reducing scattered X‐ray exposures to interventional physician operators of fluoroscopy

**DOI:** 10.1002/acm2.12801

**Published:** 2019-12-30

**Authors:** William Pavlicek, William F. Sensakovic, Yuxiang Zhou, Robert G. Paden, Anshuman Panda, Justin Hines, Sailendra G. Naidu, Rahmi Oklu

**Affiliations:** ^1^ Physics Division Department of Radiology Mayo Clinic Arizona Scottsdale AZ USA; ^2^ Physics Division Department of Radiology Mayo Clinic Arizona Phoenix AZ USA; ^3^ Department of Radiology Mayo Clinic Arizona Phoenix AZ USA

**Keywords:** fluoroscopy, interventional fluoroscopy, kinesthetic training, physician exposures, X‐ray scatter

## Abstract

Content used by Medical Physicists for fluoroscopy safety training to staff is deliverable via several formats, that is, online content or a live audience slide presentations. Here, we share one example of a kinesthetic (live, hands‐on simulation) educational program in use at our facility for some time (~10 years). In this example, the format and content specifically target methods of reducing physician operator exposures from scattered x rays. A kinesthetic format identifies and promotes the adoption of exposure‐reducing behaviors. Key kinesthetic elements of this type of training include: physician hands‐on measurements of radiation levels at locations specific to their standing positions (e.g., primary arterial access points) in the room using handheld exposure rate meters, measurement of exposure rate reduction to physicians provided by using personal protective equipment, that is, wearable aprons, hanging lead drapes, and pull‐down shields. Physician choice of procedure‐specific tableside selectable controls affecting exposure rate from optional fluoroscopy, Cine or digital subtraction angiography (DSA), along with comparative measured contribution to physician exposure is demonstrated. The inverse square exposure rate reduction to physicians when stepping back from the table during DSA is a key observation. Kinesthetic simulations in the rooms used by physicians have been found to provide the highest level of understanding giving rise to adoption of practices that are impactful for physicians. Specific training scripts are in place for physician sub‐specialization in interventional radiology, cardiology, neurosurgery, vascular surgery, and gastroenterology. This training is used for new physician staff while classroom presentations (whose content mimics in room training) are used with staff who have had previously had in room training.

## 1 INTRODUCTION

Elevated physician operator exposure from x‐ray scatter occurs primarily with specialties utilizing interventional C‐arm devices. Interventional radiologists and other specialty users are consistently and historically among the highest occupationally exposed workers in a hospital system due to their extensive use of fluoroscopic imaging and in particular the incorporation of digital subtraction angiography (DSA) and Cine acquisitions.^1^

Options exist in providing safety training education to physician staff, including an online educational self‐guided module such as a slide presentation or live learning streamed over the internet and live audience slide presentation by a qualified medical physicist (QMP).^2^ These options are widely used by hospitals as they can ensure completeness of staff as having received required education and providing documentation that this has occurred. Online options greatly facilitate scheduling of physician staff time. Less frequently used are live hands‐on kinesthetic simulation sessions as its use has impediments for implementation including physician and imaging room availability.

Kinesthetic intelligence has been separately recognized among those individuals who effectively best learn by doing.^3^ As early as 1983, published educational research has shown that various specialty training such as medical surgery is best learned by physical movement. It may be somewhat self‐selecting that those physicians who are drawn to surgery as a career choice are natively gifted with kinesthetic intelligence.^4^, ^5^ A fluoroscopy educational format that uses a live in room simulation (kinesthetic education) to a targeted audience may be well suited for the interventional x‐ray procedure physician. The added advantage of this approach is that hands‐on training simulation can be specific to the specialist physician operators of interventional fluoroscopy devices including radiology, cardiology, neurosurgery, vascular surgery, and gastroenterology. This manuscript presents content used to create kinesthetic simulations at our facility with examples of the types of physical setup and teaching points found to be helpful. An overarching theme of our curricula has been to provide the specialty physician with basic educational content focused on topics controllable by their kinesthetic movements. For example, the physician directs the positioning of protective shielding during interventional procedures. The content is very basic in its focus; these sessions do not delve into the physics of the device settings or the comparative evaluation of the numerous device features which can lower scattered x rays. These items and additional fluoroscopy safety topics are available for use in separate more conventional education sessions.

### 1.1 Kinesthetic education

Educational science has long recognized that “viewed only” content provides about 10% retention while both audio and visual content may achieve 30%–50% retention. Kinesthetic movement and actions performed by the participants achieve nearly 90% retention.^6^ With Interventional Fluoroscopy, the physician has control of the patient position on the table, movement of the table, C‐arm positions, and foot pedal engagements of fluoroscopy and acquisition (Cine and DSA) x‐ray episodes. They position themselves in a location relative to the patient that allows for the best movement of the catheter, operation of x‐radiation on and off controls, and the visualization of the image displays. It appears reasonable if not compelling to use kinesthetic education as an optional and ongoing format for operator safety in the fluoroscopy suite.

## 2 METHODS AND INSTRUCTIONAL CONTENT

An educational kinesthetic class is preferably held in the fluoroscopy room used by the physicians. In the case of interventional equipment, the availability of the procedure room for instructional purposes is often a compromise with patient care needs and staff schedules and so time may be limited. At our facility, early morning sessions prior to the first patient of the day work best. It ensures a hard stop for the conclusion of the class. To start promptly the instructor must pre‐inventory the room for available equipment, device features, and their use. Items that are noteworthy include availability of physician's personal protective equipment (PPE), table hanging lead drapes, pull‐down shields, and arm boards. Physician's PPE can be variable and found to be skirt and vest, skull caps, leaded eyewear, and added extremity protection. Differences in physician preferences exist and should be noted. It is important to map out arterial access locations (including patient positioning), physician's right and left side approaches, table height for the reference point (RP), device display air kerma rate, cumulative air kerma, and air kerma product settings and foot pedal controls. Preferred choice or most frequently used programmed fluoroscopy settings are identified (determining which fluoroscopy dose rate curves are available and actually selected for use for frequently performed procedures). As this is an interventional fluoroscopy focus training session, it is expected that procedures using cone beam CT and DSA, or Cine will be available and incorporated for class use. It is also important to note the presence of a power injector for contrast and its frequency of use with procedures. The QMP must be familiar with operating the device including modification of tableside settings, table and C‐arm movement controls, and location of tableside geometry display. Another option is to enlist the aid of an x‐ray technologist who is familiar with these device operations. The QMP must also be familiar with the movement of hanging drapes on the table and the range of movement and positioning of pull‐down shields from their storage position to their best location for physician protection with any access points used with procedures in that room. This information is incorporated into the Script for class use.

### 2.1 Handheld educational devices

A handheld ionization survey meter is needed and an independent x‐ray beam dosimetry system can be useful. A distance measurement tool is quite important. We have selected a yellow plastic cord for which three knots are created at 0.5, 1, and 2 m. This string is tied to a large metal washer to anchor the end of the string in the center of the x‐ray beam. A stack of Poly(methyl)methacrylate (PMMA) is used along with a life like head, neck, trunk, and extremity phantom. While a human like form phantom is not necessary, it has helped in quickly identifying the standing positions and measurement points for x‐ray scatter.

### 2.2 Teaching script

A prepared script serves several functions. It ensures that the desired content is included in the exercise and that this content is discussed in the desired sequence. Each participant gets a copy of this script which they use to participate in the session. If in‐room training is early in the morning before patients, a well‐scripted program can ensure completion by the scheduled time. To emphasize the kinesthetic aspect of this education, the instructor engages each participant to handle all devices as much as possible. A participant that performs actions as the class is (not surprisingly) more engaged in the topic and memory of the teaching points are facilitated.

Table 1 depicts a sample of scripted content that is handed out with Interventional Radiology physician in‐room education sessions. Here, the topic is reducing physician exposures from x‐ray scatter with fluoroscopy. The script is modified and its title altered to reflect the different physician specialty attending other sessions. Cine replaces DSA for Cardiology Catheterization procedures; left side access is emphasized with sessions for physicians involved with Heart Rhythm pacemaker implant procedures. Simulation of patient positioning is left decubitus for Endoscopy physician training. For each teaching script, it is necessary for the QMP to discuss with technologists and physicians in the specialty use of fluoroscopy to be inclusive of the salient points.

**Table 1:** A class handout Script is specific to the attendee's use of fluoroscopy, patient positioning and physician position with device access.

	Script: Physician operator exposures from X‐ray scatter fluoroscopy with interventional radiology	
	Teaching points	For: IR attending physicians, IR fellows and IR residents
1.	**Origin of scatter and C‐arm geometry** Distance of patient from tube, detector close to patient.	Setup: Patient phantom is supine with x‐ray source under the table. Detector is 10 cm above the patient. **Table Height = 15 cm below ISO center** (IRP). Standard or good geometry. Where is the origin of scatter radiation to staff? What is scatter exposure rate at 0.5 m from point of scatter at table height? What is the most frequently used fluoroscopy dose rate curve?
2.	**Bad C‐arm geometry** Bad geometry can be avoided.	Setup: Patient — supine with **25 cm Detector AIR GAP and TABLE at 10 cm below IRP (25 cm below Isocenter)** What is exposure rate at 0.5 m from point of scatter at table height? What increase in scatter x rays to physician is seen with bad geometry?
3.	**Effect of patient size/steep angles** Larger thicknesses increase scatter.	Setup: Patient is supine — 22 cm FOV and good geometry **ADD 5–8 cm PMMA**. What is the exposure rate 0.5 m at waist? Each 5–8 cm of tissue DOUBLES (or more) the scatter exposure rate! What options exist for reducing scatter with large patients? (FOV? Low dose rate fluoroscopy? Fluoroscopy pulse rate?)
4.	**Fluoro vs DSA exposure rates** Scatter and DSA — what is maximum f/s used?	Setup: Large size patient is supine — Table at IRP with 10 cm air gap 22 cm FOV (good geometry) What is the physician “guess” of what the scatter rate at 0.5 m from origin of scatter at table height? What is measured value for head, waist, and knees? What is a rough estimate of IR procedures using power injections vs hand injections? What options exist for reducing scatter exposure from DSA? (Fluoro save, use of variable DSA rate?)
5.	**Effect of stepping back** Moving to 1 or 2 m markedly decreases exposure to staff.	Setup: Large size patient supine, 10 cm air gap 22 cm FOV (good geometry) What is the participants estimate of STAFF EXPOSURE from DSA at **1.0 m** compared to 0.5 m above? What is the participants estimate of STAFF EXPOSURE from DSA at **2.0 m** compared to 0.5 m above? What physicians or other staff must be tableside with patient with DSA? Who announces that DSA will be initiated?
6.	**Effectiveness of PPE** Apron, hanging lead from table, and pull down shield are essential.	Setup: Large size patient supine, 10 cm air gap 22 cm FOV (good geometry) What is staff exposure through lead apron tableside with fluoroscopy to body? What is staff exposure through hanging lead with fluoroscopy to point at bottom of lead apron? What is the staff exposure to **head with and without** pull‐down shield? Use DSA Do hanging lead drapes and pull‐down shields locate to ALL positions used for arterial and venous access?
7.	**Quiz!: Lateral C‐arm geometry**	Set up: Large size patient supine, C‐arm with 90 lateral positioning. **Add 5–8 cm PMMA tube side verticle**. Given the choice of standing tableside, which position (patients left or right) will result in less scatter to physician? How much difference occurs in scatter rates between x‐ray tube side and image receptor side? Where is the origin of scatter?
8.	**Wrap‐up**	Summary of important choices and good practice for reducing physician exposures Good geometry, choice of pulse rate, tap and pause, stand back with power‐injected DSA. Availability of PPE for all arterial and venous access points is a must. Use of pull‐down shield is hugely important. Always use badge, placed outside of PPE at level of neck, outside of thyroid collar.

The legend organizes the content in the QMP desired presentation order of educational points. It allows for participants to interject their specific points of discussion in relation to observed levels of scattered x‐rays. Bold in the Table is the title of the corresponding teaching objective.

## 3 LEARNING OBJECTIVES FOR EACH TOPIC IN THE SCRIPT

Basic learning objectives are described below for each of the rows 1–8.

### 3.1 Origin of scatter and C‐arm geometry

The class starts with the instructor pointing to markings on the x‐ray tube housing depicting the position of the x‐ray tube focal spot as the point at which the x rays are produced. In turn, each participant confirms seeing the mark of the x‐ray tube focus and collimation. Collimation is simply depicted as forming the beam as it travels to the patient. All participants are asked to step closer to see these parts of the C‐arm and they thus start the class together in their knowledge in this elementary concept. The beam strikes the supine patient in the back of the patient and creates scattered x rays as the dominate source of all x‐ray exposure to staff in the room. Recognizing the point of intercept of the beam with the patient as the location of origin of scatter is a critical learning objective. While this location is the clearly the point from which radiation can travel to staff, these scattered x‐radiations may or may not travel through and be attenuated by the patient before striking staff. This is also confirmed using the survey meter with the participants. Exposure to a physician's knees gives higher exposure rates of x‐ray scatter than the waist or head as they stand tableside (measured values without PPE). A technologist standing at the foot end of the table is seen to not receive much exposure due to attenuation and distance as measured by a participant. The area of the beam landing on the patient is also a factor in the amount of scatter. This can be easily demonstrated by a participant using the handheld survey meter at the waist level as the collimation or FOV is changed.

Radiation that is not scattered (the primary beam) will pass through the patient, but this radiation is considerably absorbed with roughly only ~10% passing through the patient to strike the image receptor. To emphasize the importance of knowing the origin of scatter, the instructor emphasizes to the class *to remember the origin of scatter* as it will be part of the test at the end of the session as indicated in the Script.

Good geometry is depicted with a phantom on a table and the table top at 15 cm below isocenter and the image receptor no more than 10 cm above the patient and preferably closer. As the vessels in mid‐brain, neck, chest, abdomen and legs are nearly all mid‐line, the majority of interventional imaging has these vessels at C‐arm rotational isocenter so as to ensure the vessels remain in the center of the image receptor with any C‐arm angulation. This table and receptor positioning is standard practice for initial patient positioning with x‐ray technologists in preparation for physician use. The physician recognizes that good geometry with table and receptor positioning is a worthwhile learning objective; table height affects the amount of x‐ray scatter and it also relates to “work height” by the operator. In this regard, table height differences will exist and this factor is a non‐controllable or only modestly controllable behavior.^7^ For individuals of shorter stature, it is possible and important to lower the image receptor to the patient. It is useful to point out that the tableside displays of Air Kerma rate, cumulative Air Kerma, and Air Kerma Product are readily seen on the display, but these values are only applicable for a table height at the RP. With the table/receptor in good geometry, a baseline measure at 0.5 m for scattered x ray is performed and the result to be remembered for the next topic.

### 3.2 Bad C‐arm geometry

Bad geometry is depicted with lowering the table closer to the x‐ray tube than needed and raising image receptor further above the patient. As the manufacturers table side values of Air Kerma rate are not accurate with this geometry a separate dosimeter is pre‐positioned prior to the start of the class under the patient is used to note patient skin exposure. These independent values are called out by a participant. The class computes the percent increase of patient exposure as a consequence of this geometry compared to good geometry. This difference is the amount of patient dose that is often controllable and often can be reduced without affecting the procedure. The learning objective with this section is to incorporate good geometry as standard practice. The table and image receptor are returned to good geometry for the next Script topic.

### 3.3 Effect of patient size/steep angle

An anthropomorphic phantom was found to be an especially helpful addition in kinesthetic training (if available) as it allows for a concrete simulation of those specific access locations which depict the location of the physician relative to the origin of scatter. This Script has a learning objective to demonstrate the effect of larger size patients and/or thicker patient sections created with steep C‐arm angles since both cause significantly higher levels of scattered radiation. Adding 5–8 cm of PMMA to the life like patient is used to increase the x‐ray tube output to approximate the expected values for the uncontrollable variable of patient size.^8^ It is generally not appreciated by physicians that patient size markedly (not proportionately) affects the amount of scatter. Adding PMMA allows for this teaching point to be immediately appreciated. The amount of scattered x rays from the large patient to the operator physician is measured (mR/hr) by the participant and is compared to the first measurement on an average size patient. This discussion continues as the exposure to the knees, waist, and head is measured and differences in exposure at these locations are noted. The participant measurements can prompt a participant–instructor discussion of potential solutions to lessen scattered radiation to operators even with the procedures involving larger sized patients and steep angled projections. Examples of changes that are under physician control include shorter durations of step and pause, choice of reduced pulse rate fluoroscopy, and use of collimation. For example, with patients of large size and fragile vessels, the catheter is generally moved slowly.The slower movement of the catheter permits the use a reduced pulse rate (even one that introduces some image jitter) which is capable of reducing the rate of scattered x rays to the patient by 50%.^8^ If desired or used by the physician participants, an alternative arterial or venous access location (neck and/or arm) and use of left side access is additionally evaluated for differences in scattered x rays reaching these locations.

### 3.4 Fluoroscopy vs DSA scatter X‐ray exposure rates

Using the large patient size and good geometry, the amount of fluoroscopically scattered x rays at tableside at 0.5 m must always be measured and made known to the physician participants. The participants are then asked to predict an amount of scattered x rays from DSA. Generally, physicians significantly underestimate the increase in the scattered x‐ray exposure rate tableside with DSA in comparison to fluoroscopy. A measurement performed at 0.5 m tableside by a participant provides the calculation of the percent increase relative to fluoroscopy exposure rate. This demonstration has been the single most impactful moment for physician participants in our experience and is one of the primary learning objectives of the entire class. This measurement has always been strongly remembered by physicians especially by those who have held the survey meter. For physicians who use power injectors, it becomes compelling to step away from the table for these radiation events. For fluoroscopy suites which have lead drapes hanging from the table or pull‐down shields available, it decidedly motivates their routine use if handheld injections are used. The value of these behaviors, that is, stepping back and using shielding devices (including the availability of hanging tableside drapes), is determined by measurements and their value in routine use is emphasized.

### 3.5 Effect of stepping back

The participants are directed to view the washer that is seen in the center of the image. The other end of the yellow string is extended and held outwards to 2 m by a participant. The first knot at a distance of 0.5 m is quite representative of the anterior waist of a performing physician to origin of scatter when using a femoral approach. With one participant holding the far end of the string, the survey meter is held by a physician at the 0.5 m location. The instructor asks the participants what value (mR/hour) might be found at 1 m? At 1 m, only a small step back is seen as quite doable by a physician. It is our experience that physicians and other participants almost always underestimate the reduction of scattered x rays reaching 1 m. Actually performing this measurement and seeing that the distances are inarguably correct, the value of stepping back is remembered and is convincing in the value of this behavior. At 2 m, the levels are measured and a dramatic drop off in exposure shows why allied health staff are told they are quite safe at this distance. For DSA IR procedures using power injection, the physician staff can distance themselves to 2 m. It is also useful to clarify and discuss which allied health or other staff should be joining the physician at tableside if fluoroscopy or DSA is being used and the value of calling out to procedure staff the episode of use of fluoroscopy and DSA as a reminder. At our facility, we have the 2 m location from the table center identified by a change in color of the flooring as a visual aid. It is also useful to make note to the participants that the total reduction of the measured value reduction between 0.5 to 2.0 m is more than 90%.

### 3.6 Effectiveness of personal protective equipment

At a minimum, three types of protective equipment are discussed and their use clarified (and encouraged). A protective apron with thyroid shield is standard PPE for an IR fluoroscopy suite and it is worthwhile to demonstrate the protection factor provided by an apron, of course emphasizing the importance of a well‐fitted apron.^9^ This is quickly done using the handheld survey meter with participants holding the apron. The apron is shown to block scattered x rays (not primary beam) and a protection factor is estimated. Apron PPE are available in various thicknesses and the reduction in exposure rates from x‐ray scatter to physician operators can achieve 90% or can be lower with lighter weight aprons. Differences in physician preferences can be identified if desired and their use in an interventional x‐ray facility having Cine or DSA can be standardized.^9^, ^10^

Hanging tableside drapes are shown to reduce the exposure by more than 90% via measurement. It is an important pre‐procedure preparation that x‐ray technologists attach the hanging drapes as standard room preparation. Each site of access (femoral, radial, or brachial artery or internal jugular vein) should have available physician lower body protection. Room preparation also includes the movement of hanging drapes along the table. For any procedures that may be performed on the patients left side additional hanging drapes may be needed. Positioning for left femoral, arm and neck access points must be provided shielding for the physician if these access points are used. That the staff recognize that their controllable actions (provide shielding for all various access locations) are useful and impactful teaching points.

Room preparation for all procedures includes sterile draping of the pull‐down shield and its placement. For procedures, rooms that incorporate both left and right side access an overhead boom that supports the pull‐down shield as well as the table brackets for hanging drapes must be available. While 90%, 95%, or 99% attenuation factors are differences that can be measured by various PPE, the most critical point is not if a particular PPE is incrementally more protective but rather that some (and certainly those available) PPE is actually used. Pull‐down shields in particular are of huge importance as the physician's head generally is not otherwise well protected by any other means. Using a pull‐down shield that is well positioned to block scatter to the physician's head and has been consistently positioned as close as possible to fully intercept the origin of scatter for ~80% of procedures will lower the physician's head cumulative exposure by ~80%. A simple yet very worthwhile quality metric (if it can be acquired) is the frequency of use for pull‐down shields for an interventional room for each procedure.

It can be emphasized by the QMP that physician operators generally stand 0.5 m from the origin of scatter while other staff generally are positioned at ~2.0 m. The drop off in scatter to this location is ~90% or more — which is about the same reduction as an apron of 0.5 mm lead equivalence.

### 3.7 Quiz — lateral C‐arm geometry

The C‐arm sometimes is used in a lateral or near lateral projection and frequently the image receptor is found to be positioned on the patients left side with the x‐ray tube near the physician on the patient's right side with a femoral access. For the final topic and quiz, this geometry is created. To emphasize this point added PMMA is stacked vertically on edge at the table at the patient side closest to the x‐ray tube so as to create excellent scatter at the point of beam intercept. The participants are quizzed as to whether they should be standing on the left side of the patient or the right side of the patient to lower their exposure to scatter. It is again surprising to find that frequently some participants pick the image receptor side as likely to have higher scatter levels. Of course the survey meter is used to confirm the answer and the situational use of lateral projections and staff location are discussed.

### 3.8 Wrap‐up

Class duration is generally 35–40 instructional minutes not including the time allowing for participants to put on their PPE. The QMP can summarize; origin of scatter, good geometry, use of the three important PPE (especially the pull‐down shield for the physician), step and pause, and stepping back if possible — especially with power injector use with DSA. Ideally potential behavioral changes were seen to be useful and may be incorporated into routine practice.

## 4 RESULTS

The use of kinesthetic education hands on training was found impactful as it resulted in key behavioral changes actually taking place, including a dramatic increase in the use of pull‐down shields and hanging table drapes for all procedures. For example, recent review of use of pull‐down shield use averaged 90% or more which was an improvement of the initial use rate of ~25%. Badge readings of interventional radiologists were reduced ~45% in 2018 as compared to 2012. These training sessions took place in the specific rooms used by Interventional Radiologists, Cardiologists, Neurosurgeons, Vascular Surgeons, and Gastrologists. A phantom that better simulates arterial and venous accent points (or mouth access for ERCP procedures) is compelling to physicians as it correlates well with their experience and standing position relative to the patient and source of scatter.^11^ In some instances during our education session, it was found that patient left side procedures had been initiated as a clinical service but left side hanging drapes and pull‐down shields were not yet physically in place and available. Making changes to the ceiling mounted boom to support an additional pull‐down shield is costly and generally cannot be quickly added to a fluoroscopy room. Although a post education survey was not performed, the physician staff asked for repeated hands‐on classes as new staff members have joined including residents, surgical fellows, and physician assistants.

## 5 DISCUSSION

While noted above that badge readings were significantly reduced over the time frame for which all radiology physician staff received hands‐on training, it is important to note other factors. During this time (2012–2018) digital flat panel devices replaced image intensifiers, pull‐down shields were added, and individual fluoroscopy dose rate curves were optimized and standardized. To be sure multiple factors were contributory to reduced physician staff exposures over this time. Importantly, the motivation to purchase, install, and actually use pull‐down shields was generally facilitated by knowledge gained during this training. An awareness of stepping back during power injections of iodine contrast with DSA was also established.

It is also recognized that initiating a physician training program took an evolutionary route. Early on only one physician and one or two technologists were volunteers of their time to explore questions on factors which controlled their exposures. A curiosity existed for determining regions of elevated ambient exposure rates and its cause. Nursing and technologist teams did have mandatory education as did the radiology residents and now IR fellows. These educational sessions allowed for the development of a more clear picture of what is doable tableside and impactful in the hours prior to patient procedures.

For interventional fluoroscopic devices that employ DSA or Cine, the physician operator can benefit from a specialty‐specific kinesthetic training experience as it provides for adoption of key behaviors into routine practice. These behaviors are gleaned during the kinesthetic session as measurements of radiation and the utility of shielding, its positioning, and device operable parameters are comparatively evaluated and the physician exhibits preference in its use. Conclusions are drawn with practice changes that can be replicated and incorporated into daily use. In our experience, a kinesthetic format is not needed on an annual basis for individuals who have already had kinesthetic education or for which routine practices have incorporated the applicable safe practices.^12^, ^13^ Class room presentations are given annually by a QMP build on the kinesthetic training and thus generally follow similar content. See attached slide presentation content (Figs. 1, 2, 3, 4, 5, 6, 7, 8, 9, 10, 11, 12, 13, 14, 15, 16, 17, 18, 19, 20, 21, 22, 23, 24, 25, 26, 27).

**Figure 1:**
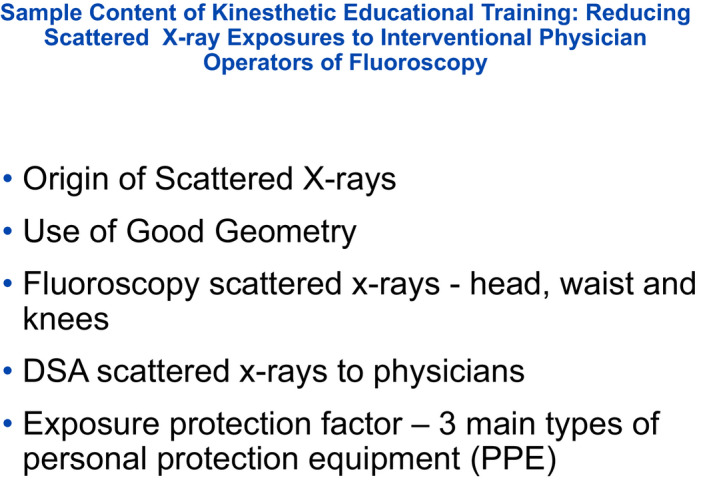
Sample content describes key objectives for hands‐on measurements and concepts that have been used with kinesthetic training. The training is limited to concepts and material that is controllable by the physician operator. Other important topics including factors that affect patient dose and scatter to allied health are not included in this training.

**Figure 2:**
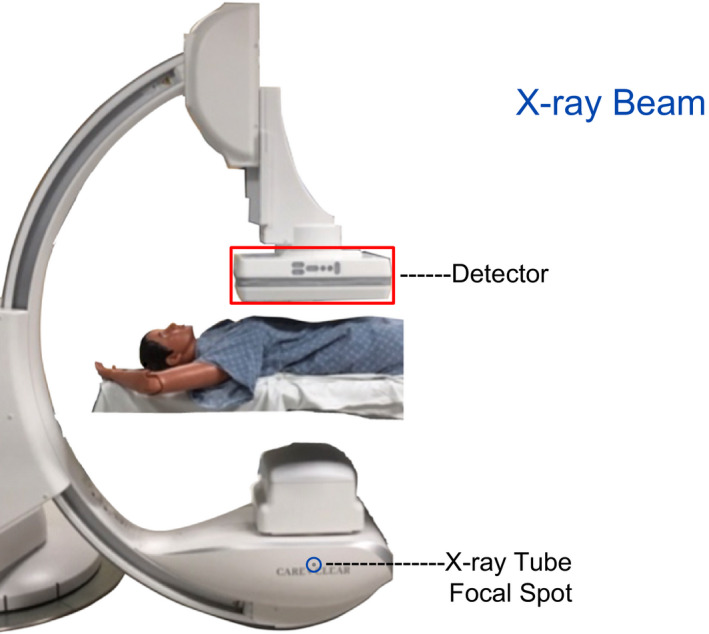
The class is joined together in visual inspection of a “dot” or marking on the tube housing which depicts the focus and the origin of x rays, the low absorbing table and pad, and the image receptor.

**Figure 3:**
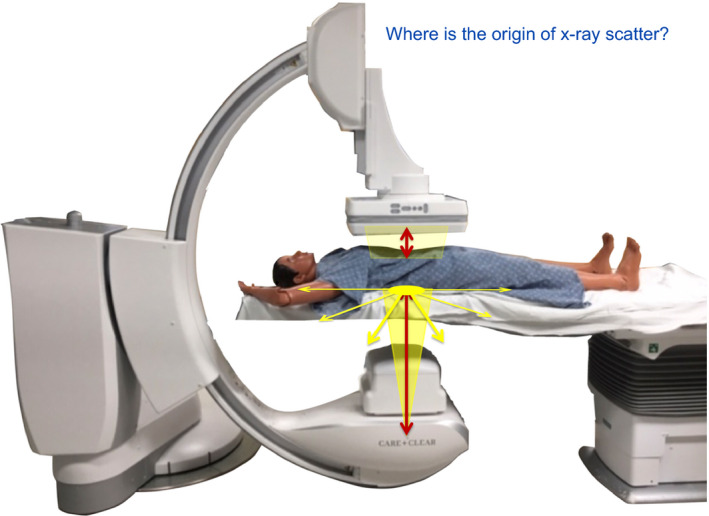
The origin of scatter is stated to be the area of the back of the supine patient struck by the x‐ray beam. The example of a flashlight beam striking the instructors hand causing large amounts of scattered light has been used as a visual.

**Figure 4:**
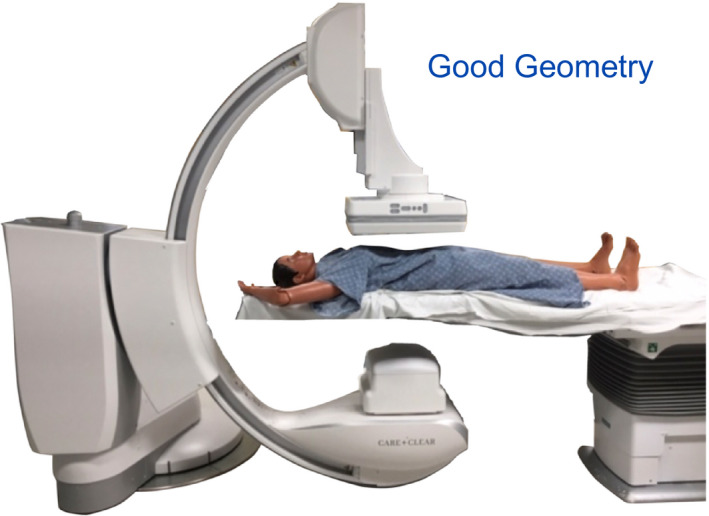
The concept of isocenter and the need to angle the C‐arm about a vessel is given, noting that the isocenter to focus is fixed while the image receptor allows movement. Having the vessel of interest at isocenter allows the image to remain in the image with C‐arm angulations.

**Figure 5:**
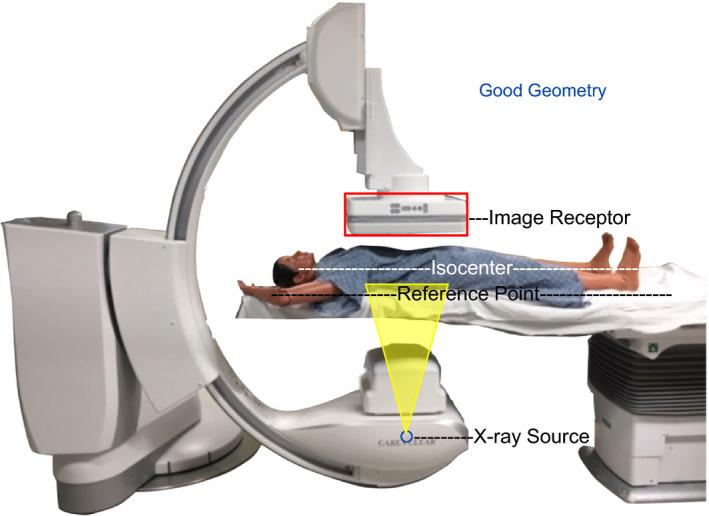
Good geometry has the vessel at isocenter which statistically has been shown for midline anatomy to have the supine patient's back at ~15 cm distance closer to x‐ray tube focus. A table height of ~15–18 cm below isocenter will roughly have the skin at the interventional reference point. Depending on manufacturer, the tableside depiction of table height having midline vessels at isocenter can be −15 or “0”.

**Figure 6:**
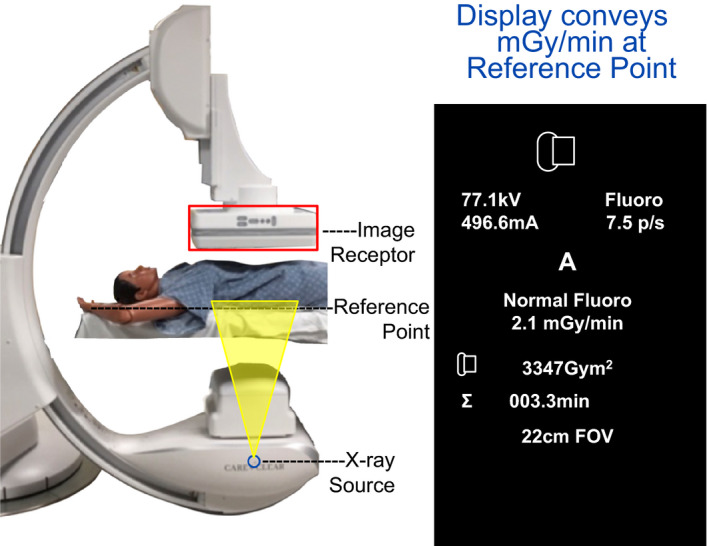
The tableside display of mGy per minute is only valid for the interventional reference point. Optionally, the SME can position an independent ionization sensor at this location to compare to air kerma at the interventional reference point.

**Figure 7:**
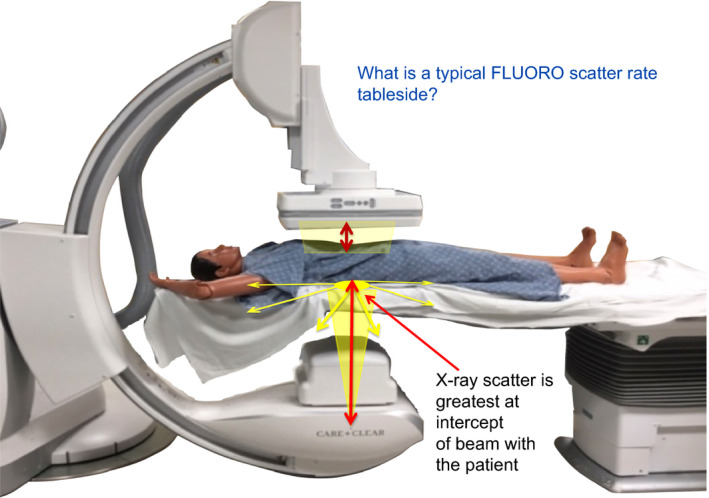
The amount of scatter created during routine fluoroscopy is measured at a typical physician positioning during catheter movement. Does 0.5 m from center of the radiation field represent a good point of measurement — one that approximates distance to the physician?

**Figure 8:**
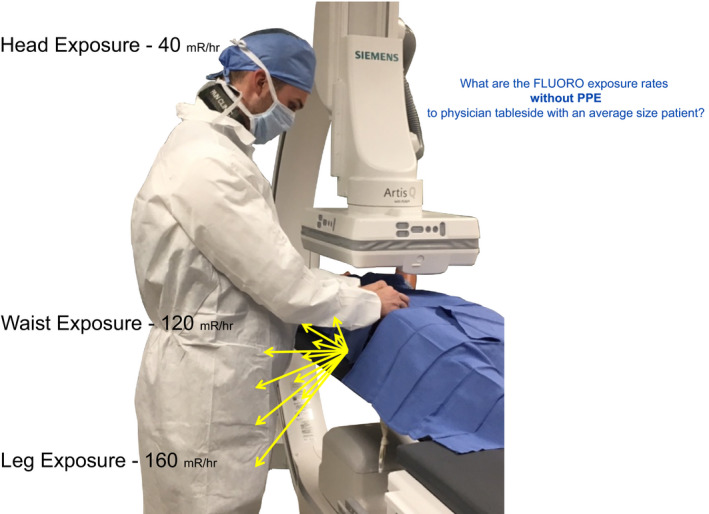
How do measured exposure rates differ at physician's knees, waist, and head?

**Figure 9:**
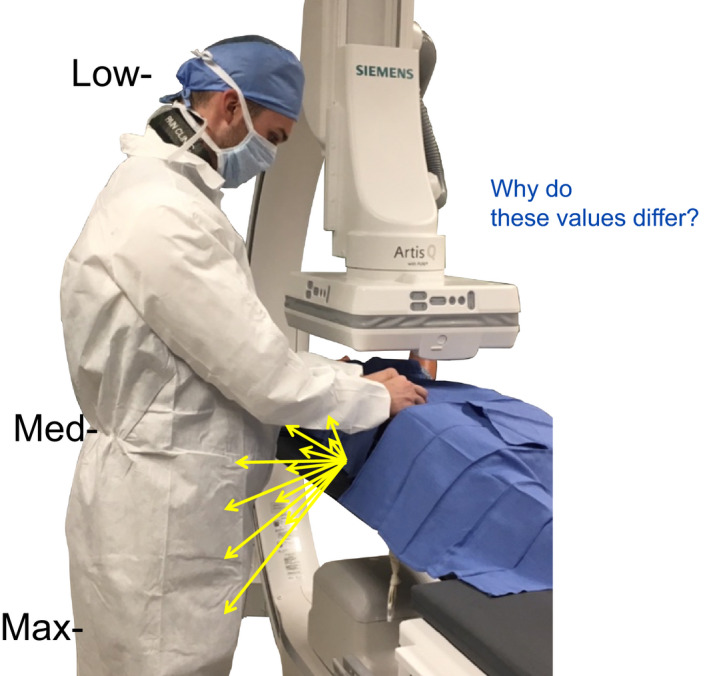
The amount of scatter created during routine fluoroscopy is measured at a typical physician positioning during catheter movement. What factors cause these differences?

**Figure 10:**
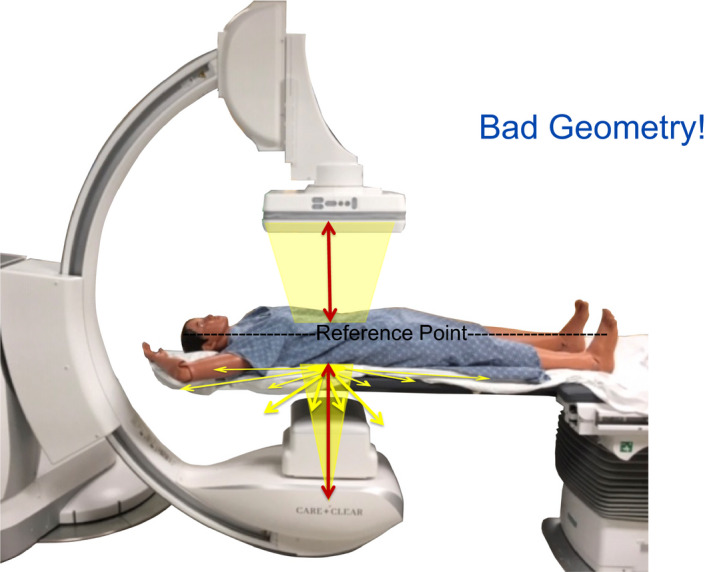
Lower table 10–15 cm closer to the x‐ray tube. Elevate the image receptor 10 cm. This extreme example is not intended to show a typical occurrence but to emphasize what occurs with a bad geometry. In this example, exposure to waist nearly doubles. Fig. 12 concludes with the theme of controlling what is possible.

**Figure 11:**
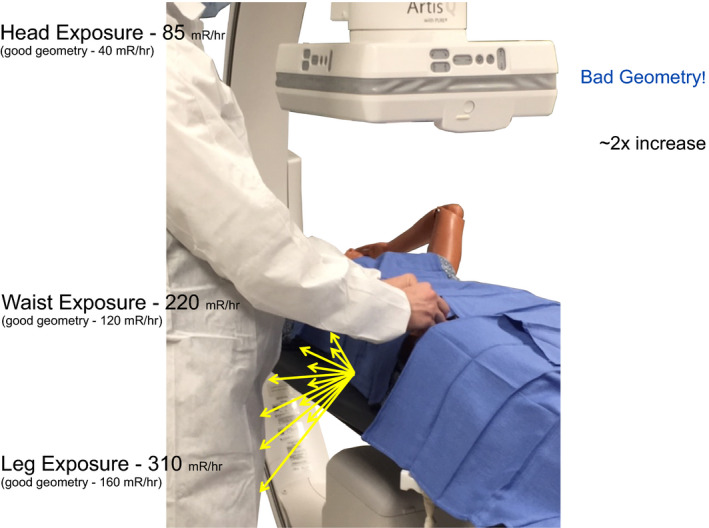
What are the measured values of scattered x rays with bad geometry?

**Figure 12:**
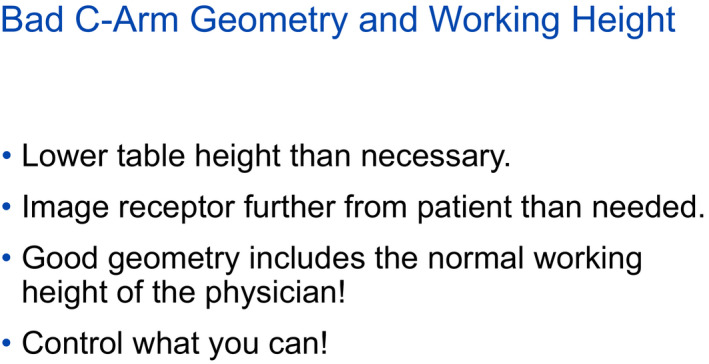
Table and image receptor positioning are controllable factors that affect the amount of scattered x rays.

**Figure 13:**
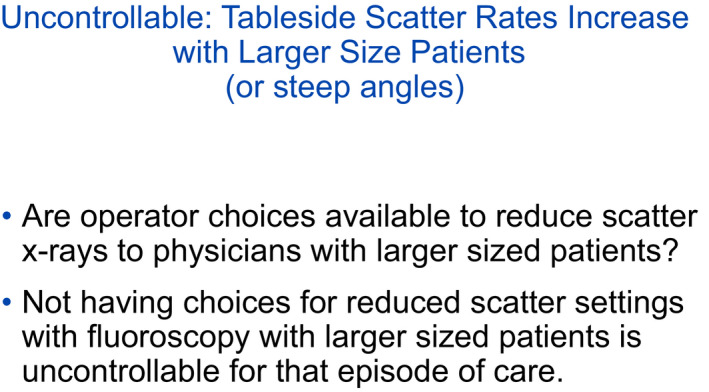
While table and image receptor positioning are controllable, patient size and the need for angled C‐arm projection are not controllable (along with physician height). The addition of 5–8 cm of Poly(methyl)methacrylate PMMA is used to create large attenuation with good geometry. It is usually underappreciated the impact of patient size (and steep angles) has with respect to amount of scattered x rays.

**Figure 14:**
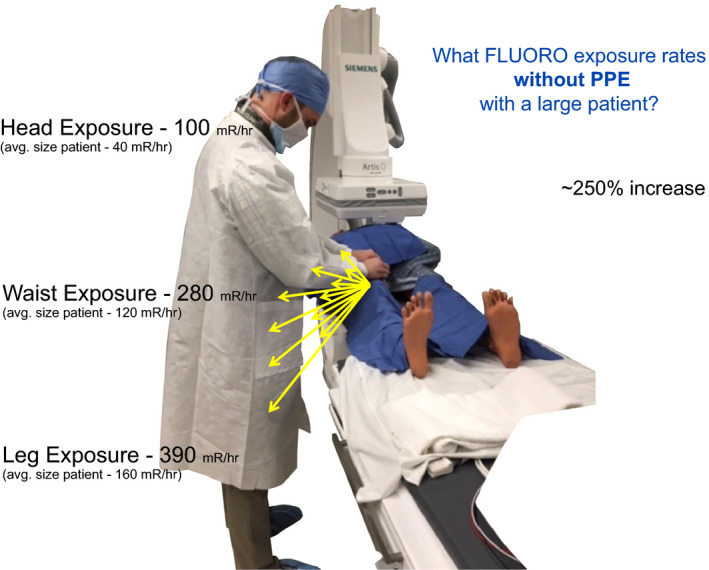
In this example, a 250% higher than an average sized patients scatter was observed. While uncontrollable, a large‐sized patient could have device settings or choices that may mitigate the amount of scatter. What would be some options that would be helpful to lower the scattered x rays?

**Figure 15:**
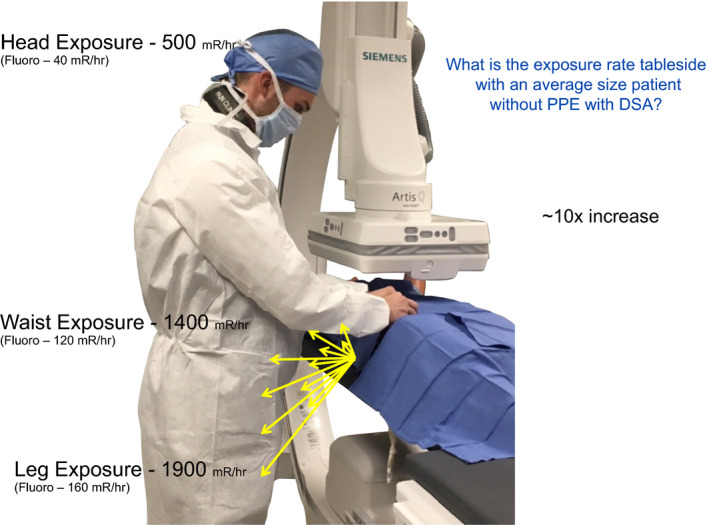
The participants are asked what digital subtraction angiography (DSA) rate is commonly used and DSA is then engaged with good geometry with the larger sized patient. DSA levels are measured at tableside (0.5 m at waist level). What number of DSA acquisitions is commonly repeated? Is power injection for DSA available and how frequently is it used? DSA tableside levels of scattered x rays with large patient simulation have been the most impactful educational point. Stepping back with power injections and reduced fluoroscopy pulse rate with larger sized patients has been adopted as practice.

**Figure 16:**
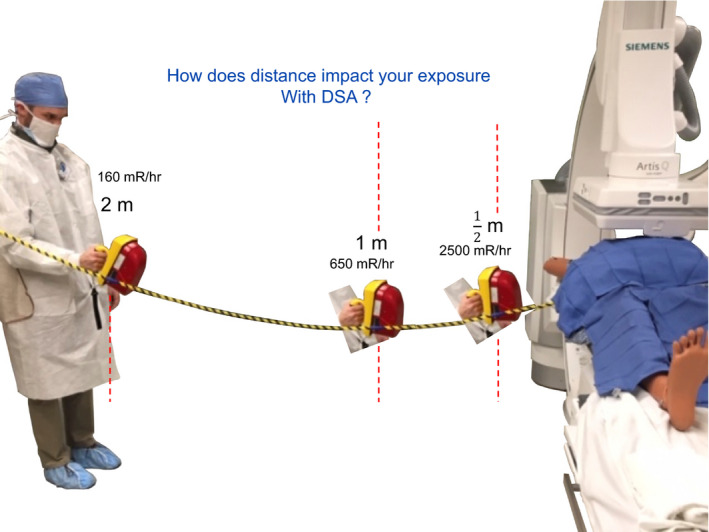
Using digital subtraction angiography (DSA), a measurement at tableside is taken and the participant is challenged to estimate what would be the level if a step back to 1, 2 m is taken? The measured values are close to following the inverse square law and give confidence to behaviors that even marginally increase distance from the origin of scatter.

**Figure 17:**
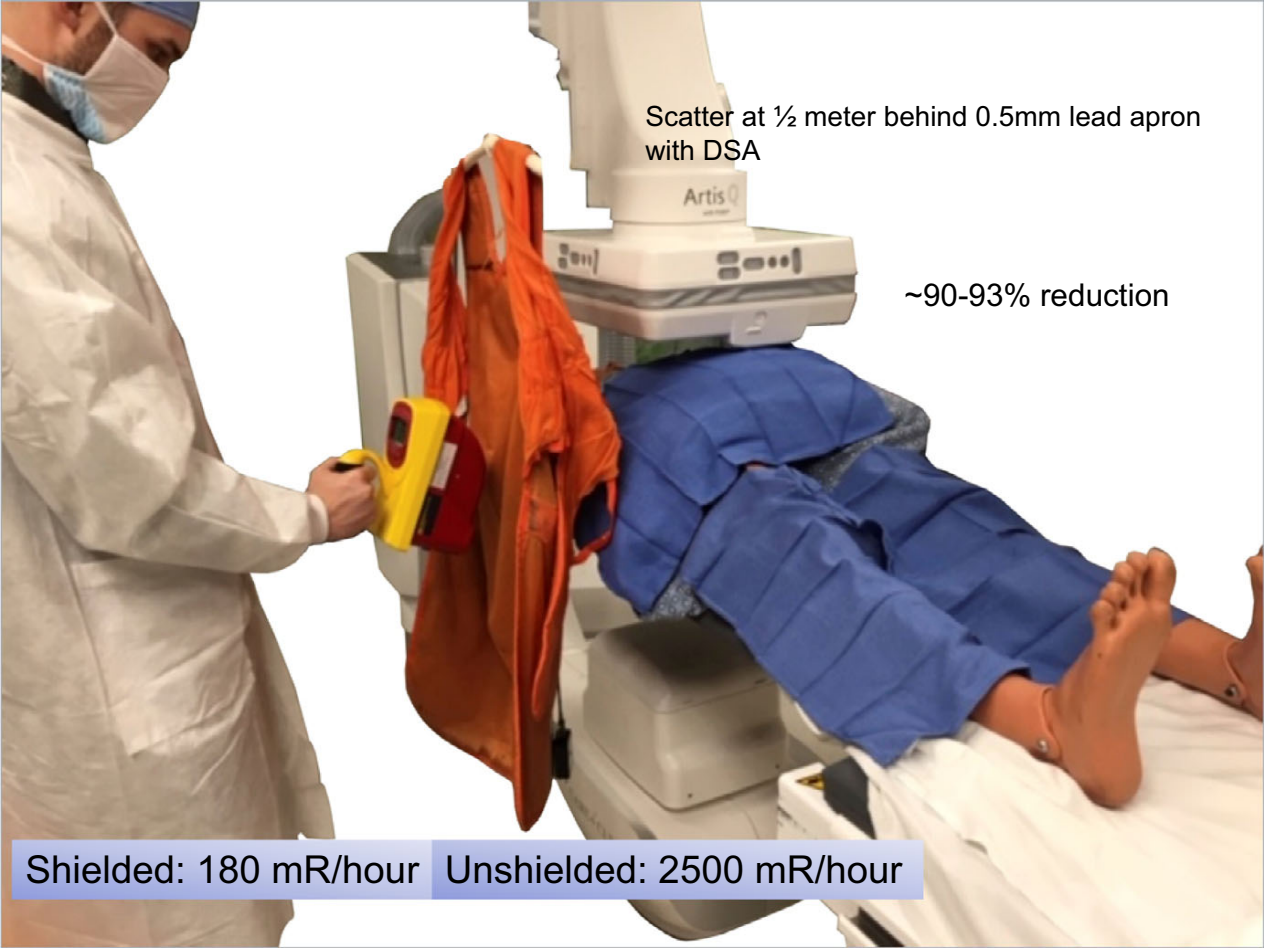
The protection afforded by the lead apron personal protective equipment (PPE) is quickly made, and questions arise as to options with specified lead equivalence and weight/benefit advisement.

**Figure 18:**
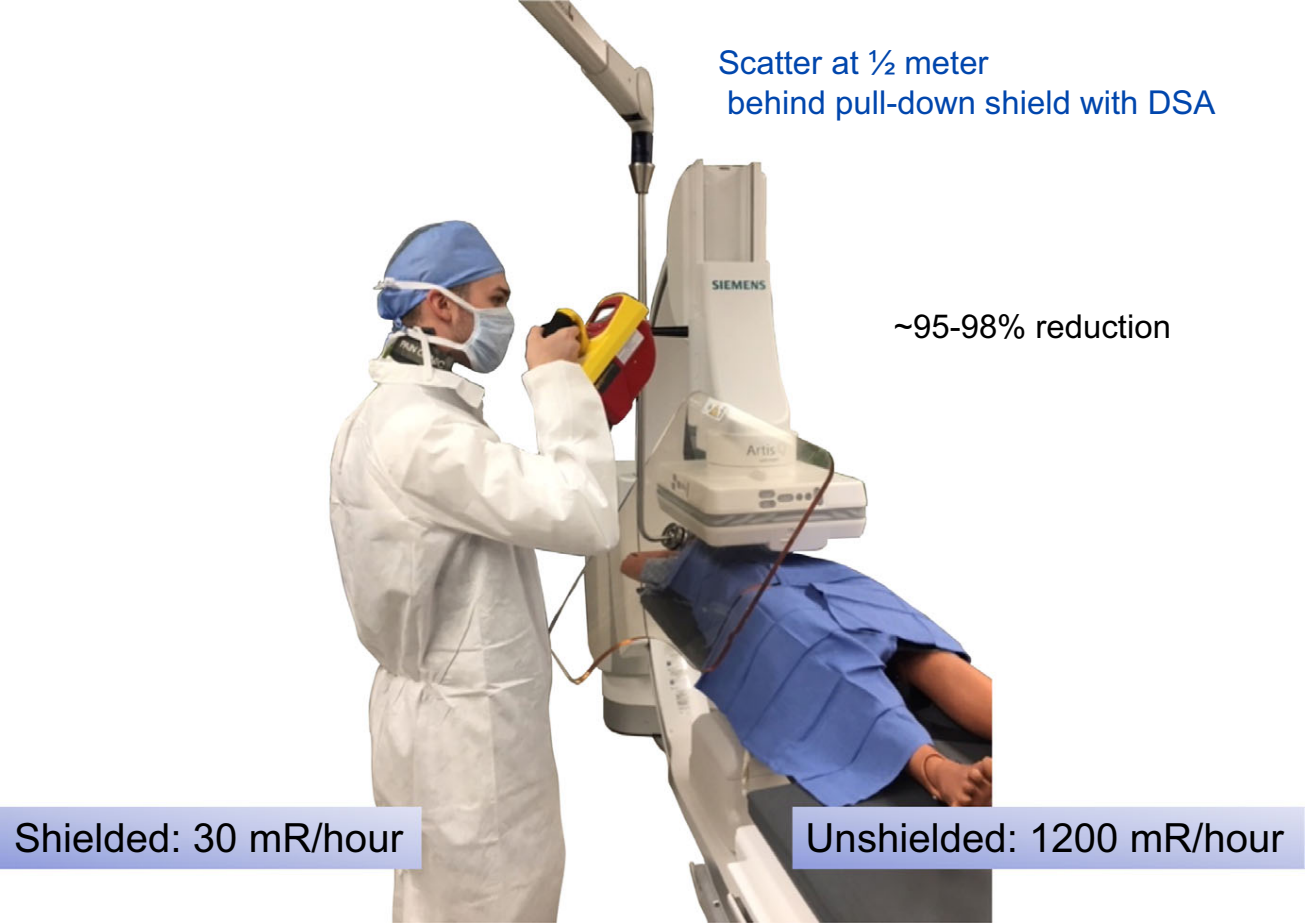
The protection afforded by the pull‐down shield is another most important demonstration. Usually, other methods do not exist for protection of the physicians head. A pull‐down shield must be mechanically available for the attending physician protection for all points of arterial access, both left and right side of patients. The articulation of the shield must be understood by technologists for room preparation. The question can be asked if a pull‐down shield would be used if the room preparation would include its prepositioning. For physicians standing tableside with hand injection during digital subtraction angiography (DSA) on a large patient, the scattered x‐ray values can be measured again.

**Figure 19:**
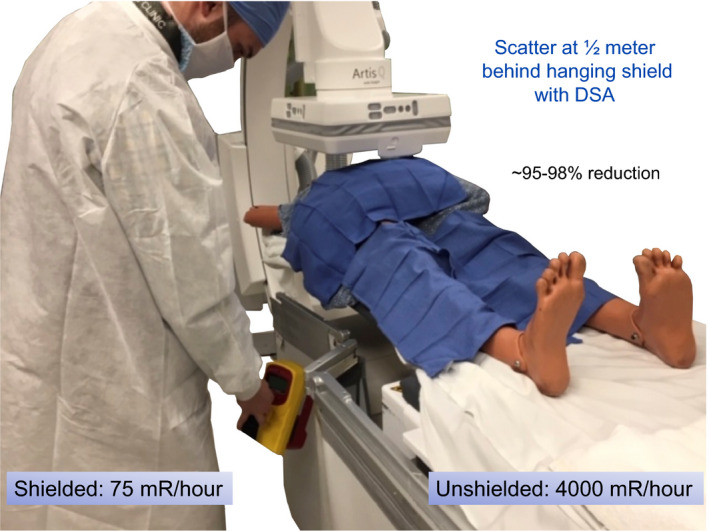
The protection factor afforded by hanging drapes is made. Prepositioning of the drapes for the location of access is needed for both left and right side of the patient. Table rails will differ and may require familiarity by technologist staff.

**Figure 20:**
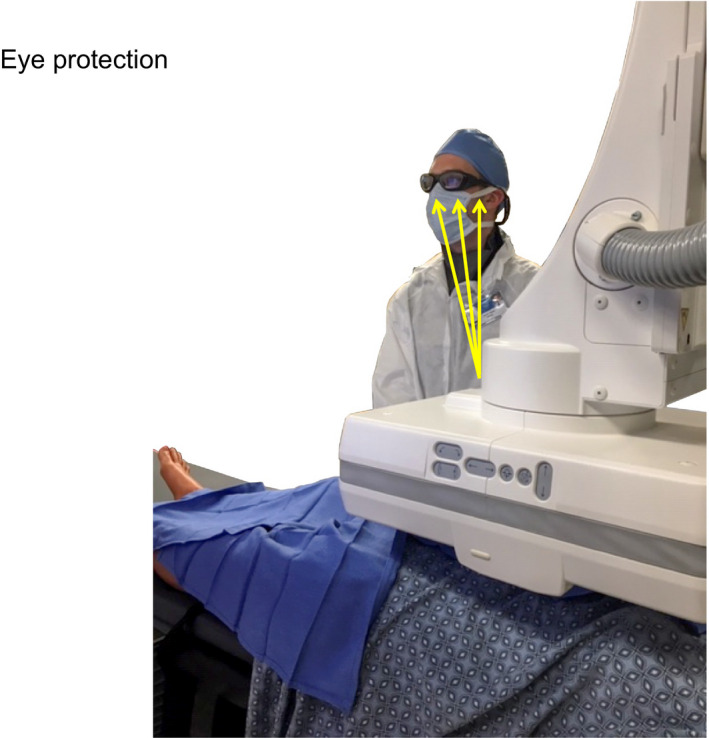
A physician may use leaded eyewear for fluoroscopy and digital subtraction angiography (DSA) imaging. A measurement can be made at the face of the physician with normal display monitor viewing.

**Figure 21:**
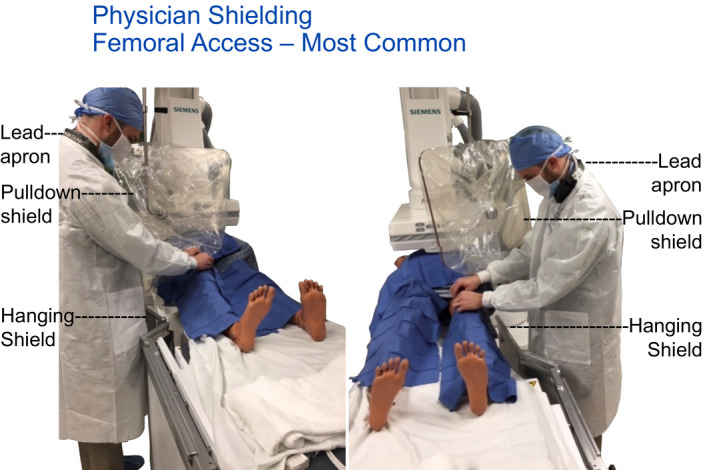
A high frequency procedure involves the physician on the patient's right side using femoral access. All three shields are demonstrated in this example of best practice and are also used with patient's left side access in this room.

**Figure 22:**
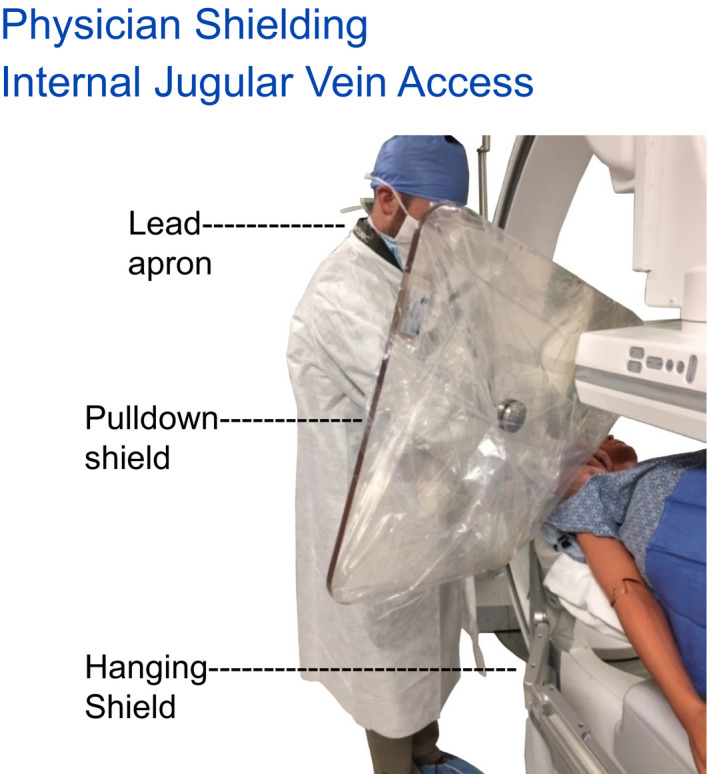
A pull‐down shield is moved to provide protection for right internal jugular vein access. Prepositioning would be needed.

**Figure 23:**
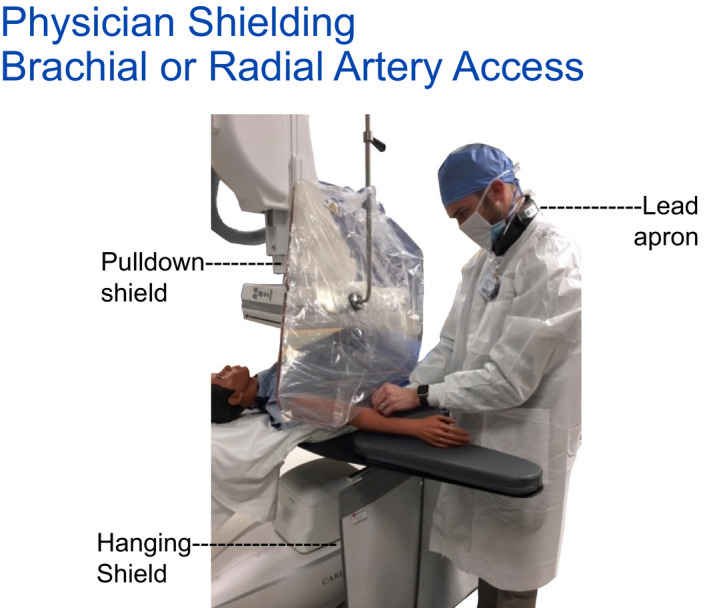
A pull‐down shield is moved to provide protection for left brachial or radial artery access. Prepositioning would be needed.

**Figure 24:**
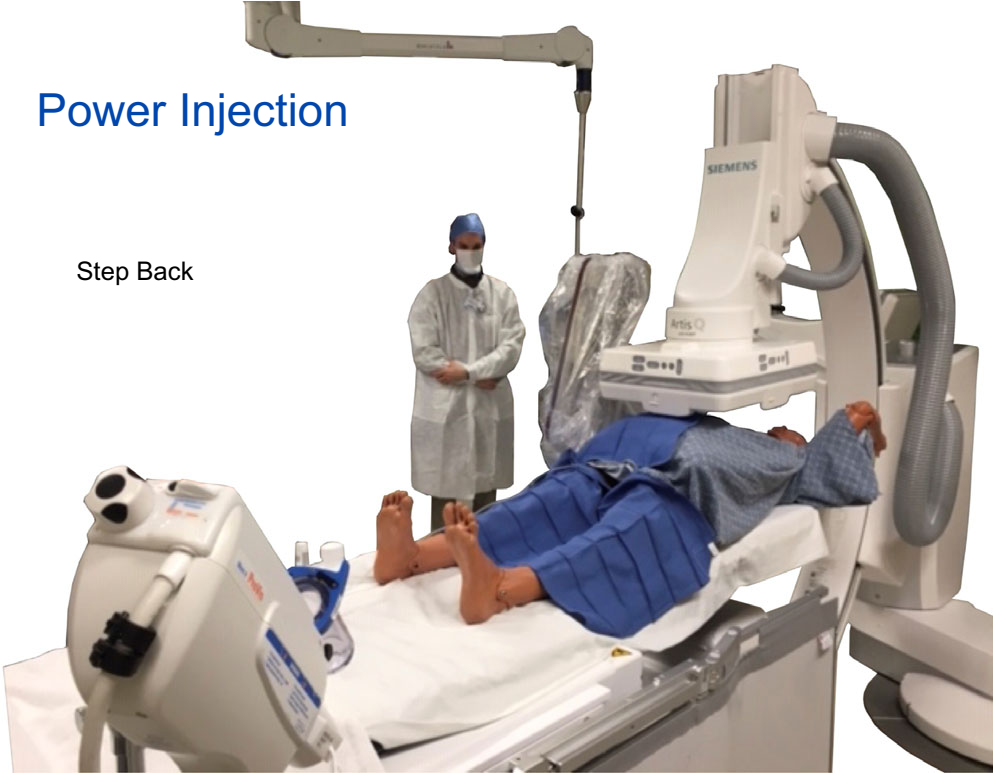
An example of stepping back with power‐injected digital subtraction angiography (DSA) is given.

**Figure 25:**
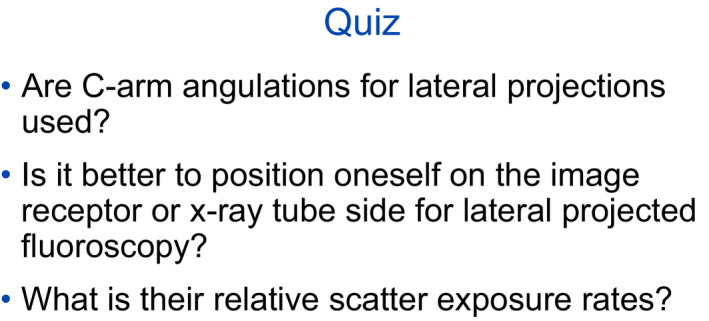
The “kinesthetic” quiz is posed as to where to stand (left or right side of patient) obtain lower scatter with lateral projections of the C‐arm. Each participant can give their choice. Vertically stacked added Poly(methyl)methacrylate (PMMA) is used to create ideal scatter conditions.

**Figure 26:**
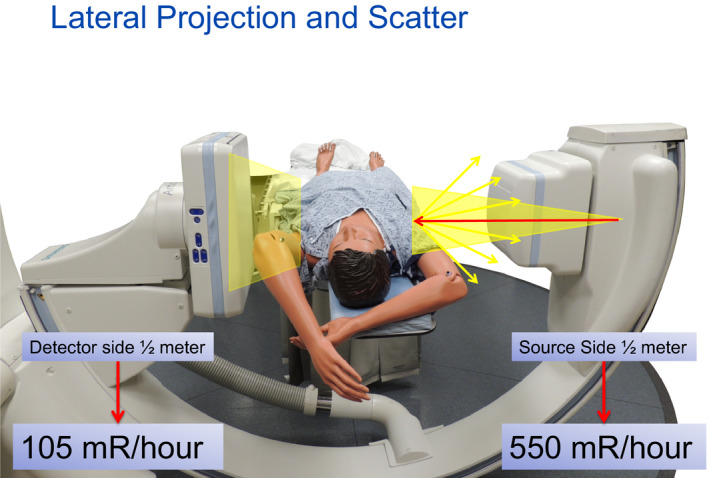
A participant who may be unsure is asked to measure both sides of the table.

**Figure 27:**
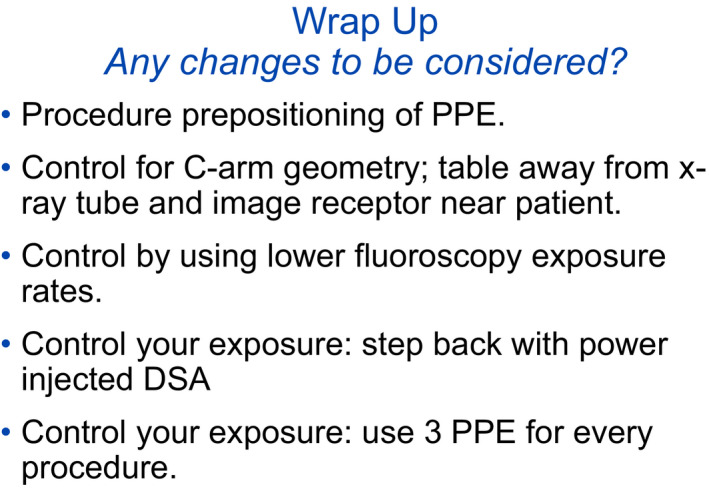
The wrap‐up is a key part of the session. What are the items which can be taken forward and are worthy of follow on discussion with the goal of integrating into clinical practice as a standard?

Please note the values in the attached slides made with the survey meter are in units of mR/hour. This is not consistent with SI units (uSv/hour) for surveys or mGy/min given by all fluoroscopy devices. Generally the participants accept the meter values as the training is focused on measurements of x‐ray scatter.

## 6 CONCLUSION

A kinesthetic education format was developed and has been found to be impactful for improving operational safety of the physician staff — arguably the best testimony a measure of education quality. Physicians gained insights and recognition of the value of adopting controllable best practice behaviors. The key behaviors of table and receptor positioning, suggestions for dealing with the high exposure rates found with larger sized patients, understanding the dose rates associated with DSA relative to fluoroscopy and the value of stepping back if possible were demonstrated. While the session uses the term “controllable behaviors”, a kinesthetic education format whose content is aligned for each physician specialty was anecdotally found to be highly effective as compared to generalized educational formats using online self‐directed modules.

## CONFLICT OF INTEREST

William F. Sensakovic is founder of Telerad Physics Teaching, LLC. All other authors declare that having no conflicts of interest.
